# Long‐term cooperative relationships among vampire bats are not strongly predicted by their initial interactions

**DOI:** 10.1111/nyas.15241

**Published:** 2024-10-27

**Authors:** Gerald G. Carter, Simon P. Ripperger, Vi Girbino, M. May Dixon, Imran Razik, Rachel A. Page, Elizabeth A. Hobson

**Affiliations:** ^1^ Department of Evolution, Ecology and Organismal Biology The Ohio State University Columbus Ohio USA; ^2^ Smithsonian Tropical Research Institute Balboa Ancón Panamá; ^3^ Museum für Naturkunde Leibniz‐Institut für Evolutions‐ und Biodiversitätsforschung Berlin Germany; ^4^ Department of Biological Sciences University of Cincinnati Cincinnati Ohio USA

**Keywords:** cooperation, *Desmodus rotundus*, helping, social bonds, social networks

## Abstract

In many group‐living animals, survival and reproductive success depend on the formation of long‐term social bonds, yet it remains largely unclear why particular pairs of groupmates form social bonds and not others. Can social bond formation be reliably predicted from each individual's immediately observable traits and behaviors at first encounter? Or is social bond formation hard to predict due to the impacts of shifting social preferences on social network dynamics? To begin to address these questions, we asked how well long‐term cooperative relationships among vampire bats were predicted by how they interacted during their first encounter as introduced strangers. In Study 1, we found that the first 6 h of observed interactions among unfamiliar bats co‐housed in small cages did not clearly predict the formation of allogrooming or food‐sharing relationships over the next 10 months. In Study 2, we found that biologger‐tracked first contacts during the first 4–24 h together in a flight cage did not strongly predict allogrooming rates over the next 4 months. These results corroborate past evidence that social bonding in vampire bats is not reducible to the individual traits or behaviors observed at first encounter. Put simply, first impressions are overshadowed by future social interactions.

## Graphical abstract

Why do particular groupmates form bonds? When social relationships are based on immediately observable traits, social bond formation might be predictable from initial interactions. However, when social bonding is shaped by a history of interactions, the resulting bonds may be unpredictable. In vampire bats, long‐term cooperative relationships were not strongly predicted by first interactions. First impressions appeared to be overshadowed by social history and changes in social networks.

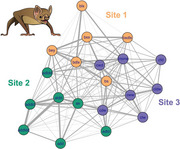

## INTRODUCTION

In many group‐living mammals, the health, survival, and reproduction of individuals are enhanced by their success in forming strong and stable affiliative social relationships or “social bonds.”^1^, ^2^ Social bonds can help animals to avoid conflict, gather information, access resources, and receive help in times of need,^1^, ^2^, ^3^, ^4^, ^5^, ^6^ yet it remains largely unclear why particular pairs of groupmates form social bonds and not others.

Is social bonding predictable from the immediately observable traits of two individuals? One way to address this question is to test if later social relationships are predictable from first interactions. Just as dominance relationships in some species can be predicted from body size,^7^ social bond formation between two individuals might be largely a predictable outcome of their immediate phenotypic cues. Two groupmates might be highly likely to form a social bond because they have similar scents, behaviors, or personalities.^8^, ^9^, ^10^ If social bonds are relatively predetermined by similarity of scents or other immediately observable traits, then how two animals interact during their first encounter should predict their long‐term relationship outcomes. We call this the *immediate assessment hypothesis*.

Most behavioral traits, however, are not immediately observable. The assessment of personality traits like boldness, activity, and exploration requires sampling over time.^11^ Moreover, the expressions of social personality traits like aggressiveness, gregariousness, and cooperativeness are influenced by both actors and receivers. The greater the importance of such traits and the harder they are to assess, the less we should expect first impressions to reflect long‐term relationship outcomes.

Furthermore, most concepts of social bonding assume that the process is shaped not only by the behavioral traits of each individual, but also by changes over time in their dyadic relationship and in the larger social environment, that is, the “market” of all possible relationships. For example, a highly aggressive individual might become more tolerant only toward a particular groupmate, and this behavior might change again due to the addition of a third individual to the group. In these more socially complex cases, dyadic relationships emerge from social preferences that partners form by somehow integrating social experiences over time and by comparing across multiple relationships.^12^ Just as social dominance can be shaped by experiencing wins and losses with the same or different partners,^7^ two individuals might socially bond or not depending on the relative quality of their current relationships versus their other options of possible partners.^5^, ^13^, ^14^, ^15^ Social history is also influenced by nonsocial factors such as shared experiences or use of space.^16^, ^17^, ^18^ If social bonding depends on the integration of unpredictable social and nonsocial events over time and across social options, then the initial interactions between two strangers should have little power for predicting long‐term social bonds. In other words, first impressions would be overshadowed by the history of social interactions. We call this the *social history hypothesis*.

The immediate assessment and social history hypotheses are actually labels for different ends of a continuous spectrum representing the predictability of social bond development. On one end, social bonds are highly repeatable and predictable because they are caused by the relatively fixed traits of individuals. On the other end, they are shaped in complex ways by a dynamic social history. An important long‐term challenge of understanding social integration is to model and measure how the relative roles of individual traits and social history differ across relationships and time. However, given the rarity of data on the formation of new long‐term cooperative relationships, testing these two hypotheses is an important first step.

Here, we tested the immediate assessment hypothesis in female common vampire bats (*Desmodus rotundus*). Female vampire bats that meet as strangers can form cooperative relationships that involve two cooperative investments: allogrooming and regurgitated food sharing.^4^, ^18^, ^19^, ^20^, ^21^, ^22^ In two studies, we analyzed the first social interactions between experimentally introduced unfamiliar female common vampire bats, some of which later developed long‐term cooperative relationships that varied in strength.^18^, ^19^ In Study 1, we asked whether the strength of long‐term cooperative relationships was clearly predictable from the initial interactions among bats introduced within small cages. After finding that contact duration was a proxy for affiliative interactions, we asked in Study 2 whether the strength of long‐term cooperative relationships was clearly predictable from initial contact durations among bats introduced in a large flight cage and tracked with proximity sensors.

## STUDY 1: ARE COOPERATIVE RELATIONSHIPS PREDICTED BY INITIAL INTERACTIONS?

### Methods

Our first study tested the immediate assessment hypothesis by combining new observations and analyses of first interactions with a published dataset of the later cooperative relationships that formed among the same individuals. The published dataset included rates of allogrooming and regurgitated food sharing based on 638 h of observation from a 15‐month study on how new food‐sharing relationships form between vampire bats.^19^ During this published study,^19^ adult females were caught from two distant sites in Panamá and housed in two separate groups. Although we use the term “unfamiliar bats” for pairs of bats from different sites, the unfamiliar vampire bats could possibly hear each other's calls but not physically interact prior to introduction. During the controlled introduction phase, one bat was introduced to 1–3 unfamiliar individuals in small cages (28 × 28 × 40 cm^3^),^19^ which ensured that strangers interacted within each of 18 small groupings of 2 or 4 adult female bats per cage (3 females had pups attached to them). Small cages were video recorded with infrared surveillance cameras. Next, during the mixed‐group phase, all bats were allowed to freely interact for over 10 months in an outdoor flight cage at the Smithsonian Tropical Research Institute in Gamboa, Panama. The size of the flight cage (2.1 × 1.7 × 2.3 m^3^) ensured that bats could either interact or avoid each other.

#### Long‐term cooperative relationships

Cooperative relationships in vampire bats are reflected by rates of allogrooming and food sharing.^20^, ^21^, ^22^, ^23^ During both phases of the experiment described above (controlled‐introduction phase and mixed‐group phase), Carter et al. ^19^ induced allogrooming and food sharing by isolating and fasting individual bats for 24 h then putting them back in their cage for 1 h. Observers then scored food sharing and allogrooming with the focal bat from video‐recorded “focal follows”^24^ that each lasted 1 h (controlled‐introduction phase = 106 focal follows, mixed‐group phase = 532 focal follows). After each focal follow, focal bats were fed in isolation to prevent food sharing outside the observation time. For further details, see Ref. 19.

#### First interactions

To obtain new data on unfamiliar bats’ initial interactions, we found all cases where a stranger bat was placed in each small cage during the controlled introduction phase and the subsequent interactions were captured on infrared surveillance video (24 bats, 35 introduced pairs). These first hours of initial interactions had not been previously observed and analyzed. An observer scored the first 6 h of behavior, including the onset and duration of all events of aggressive interactions (e.g., hitting with thumbs, pushing, biting, or baring teeth) and affiliative interactions (sniffing or licking, which were combined due to the difficulty of delimiting these behaviors). To avoid bias, the observer was blind to the long‐term relationship outcomes between the bats. Due to the death of four bats, we only had long‐term allogrooming and food‐sharing relationships (in both directions) for 26 of the 35 pairs where initial interactions were video recorded (20 bats, 26 introduced pairs).

#### Data analysis

To test whether first interaction rates predicted long‐term allogrooming and food‐sharing rates, we used a permutation‐based approach to testing the null hypothesis. We chose an approach that made minimal assumptions about the data‐generating process because the first interactions were a sample of highly structured and non‐independent data. In particular, estimating a covariance matrix for unbiased effects of actors and receivers requires that all pairs have an opportunity to interact, ideally in isolated pairs,^25^ but in this dataset, only some bats were placed together, some bats were introduced to one individual, and others were introduced to several. We therefore created a custom null model that controlled for these sampling biases and then compared an observed statistic (the association between first interactions and long‐term cooperative relationships) to those statistics expected from the null model.

To do this, we first fit an aggregated binomial generalized linear model for each combination of one predictor (either aggressive or affiliative interactions) and one response (either long‐term allogrooming or food sharing rates), leading to four models. To ease comparison, we scaled and centered the predictors (seconds of either affiliative or aggressive first interactions). We modeled the response (long‐term allogrooming or food‐sharing rates) as the total possible seconds with and without the behavior (e.g., the mean probability of grooming per possible second of observation) over a period of 303 days.^19^ Observation times for dyadic food sharing ranged from 240 to 960 min per pair (mean = 784 min), and observation times for dyadic allogrooming ranged from 540 to 1860 min per pair (mean = 1567 min). Allogrooming and food sharing were only considered possible when both actor and receiver were present during the observation and when one bat was being focal followed, and food sharing was only considered possible when the receiver was also fasted. We extracted the predictor coefficient from each model as an observed value.

Next, we estimated the expected values of coefficients under the null hypothesis that introduced bats directed their behaviors randomly to the 1–3 other bats within the small cages. To do this, we randomly permuted the interactions that could have been observed within the small cages under this null hypothesis (5000 randomizations). We fit the same models to the 5000 randomized datasets to estimate the distribution and 95% quantiles of expected coefficients. We calculated one‐tailed *p*‐values as the number of expected coefficients that were equal to or more extreme than the observed values. We used one‐tailed *p*‐values because we predicted that affiliative first interactions would predict stronger cooperative relationships and aggressive first interactions would predict weaker ones. To estimate precision in the frequency of interaction types, we used binomial tests to calculate 95% confidence intervals (95% CIs). All analyses were done in R.^26^

### Results and discussion

Introduced female vampire bats from different capture sites were about 10 times more likely to have affiliative interactions than aggressive ones. Across 35 pairs of bats, 30 pairs engaged in affiliative interactions (86%, 95% CI = [70%, 95%]) and only 3 pairs engaged in aggressive interactions (8.6% [1.8%, 23%]). Across the 70 directed actor–receiver pairs, 52 actor–receiver pairs had affiliative interactions (74% [62%, 84%]) and 3 actor–receiver pairs had aggressive interactions (4.3% [0.9%, 12%]).

**FIGURE 1:**
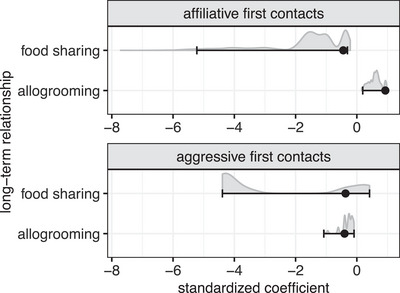
No clear evidence that initial affiliative or aggressive interactions predicted long‐term cooperative relationships. Plots show the observed statistical relationship (black circle) and expected statistical relationship under the null hypothesis (gray shading: probability distribution, black error bars: 95% quantiles) describing how long‐term food‐sharing or allogrooming relationships are predicted by interactions at first contact that were either affiliative (top panel) or aggressive (bottom panel). Observed values within the error bars show that first contacts did not predict long‐term food sharing or allogrooming relationships significantly better than chance.

We found no clear evidence that either aggressive or affiliative interactions during first encounters predicted long‐term allogrooming or food sharing rates (Figure 1). All four of the observed statistical associations between predictors and responses occurred with greater than 5% frequency when the null hypothesis was true (Figure 1, Table S1).

Although these results suggest that first interaction times do not clearly predict long‐term cooperative relationships, they are based on only 26 introduced pairs, and we did observe a trend for affiliative interactions predicting long‐term grooming (Figure 1, Table S1). A downside of not having sampled all possible pairs is the lack of a model allowing for an interpretable effect size, especially given the high prior probability that the initial interactions have an association with long‐term cooperative relationships that is greater than precisely zero. In Study 2, all pairs were able to freely interact, which allowed for a better estimation of both individuals as random effects, and hence a better estimation of an effect size. In Study 2, we also used contact duration as a proxy for initial affiliative interactions, because in Study 1, the average duration of an affiliative interaction (mean = 252 s [143, 384]) was 10 times longer than the longest observed aggressive interaction (25 s).

## STUDY 2: ARE COOPERATIVE RELATIONSHIPS PREDICTED BY INITIAL ASSOCIATIONS?

### Methods

In our second study, we tested whether long‐term allogrooming relationships among vampire bats were predicted by initial rates of contact, measured via proximity sensors. We again used new data from proximity sensors to predict allogrooming relationships estimated from published data.^18^ The published data were from another experiment that measured and manipulated the formation of allogrooming relationships among unfamiliar female common vampire bats from three distant roost sites, including the two roost sites used in Study 1.^18^ None of the bats used in data analysis for Study 2 were used in data analysis for Study 1.

In addition to testing our main hypothesis, we also tested our prediction that bats would show ingroup/outgroup biases in association such that the familiar bats (captured from the same wild site) would spend more time in proximity than would unfamiliar bats, consistent with related past findings.^19^, ^22^, ^23^, ^27^ At the network level, this effect would mean that as network density increased (greater contact among bats), so would assortativity based on familiarity (greater clustering of familiar bats).

#### First contacts

To track proximity, we glued a proximity sensor to each bat's back immediately prior to their introduction into an outdoor flight cage (2.1 × 1.7 × 2.3 m^3^). We used Osto‐Bond skin bonding latex adhesive (Montreal Ostomy Products) to attach the devices to the dorsal fur. Past studies using the same glue and attachment method show that vampire bats mostly stop trying to remove the tag within the first hour (the proportion of time they spend scratching at the tag declines from 53% to 13%), and that they appear to habituate to these tags within 4 h.^28^

We defined “contact” as encounters between sensors in which the maximum received signal strength during the encounter was within the top 5% of observed values. In past studies, long‐term allogrooming and food‐sharing relationships were correlated with daily proximity networks based on the top 1%–10% of signal strength values, which correspond to distances between the sensors of about 2 and 50 cm, respectively.^21^, ^23^

We defined “first contacts” as contacts among bats that were previously unfamiliar (i.e., bats from different capture sites that were never in physical contact before attachment of the proximity sensors). We introduced the 20 sensor‐tagged female vampire bats into the flight cage at 2:15 a.m., and we measured the durations of first contacts among them (190 pairs) for the first 4, 8, 12, 16, 20, and 24 h after being introduced together into the flight cage. We chose these times to create linearly increasing time periods that matched expected patterns of behavior throughout the day and night. Specifically, from hours 0–4 (02:15 to 06:15), bats were likely to be active in the flight cage (before sunrise at 06:00). From hours 4–16 (06:15 to 18:15), the bats were likely to be roosting in one of three roost boxes and possibly sleeping (before sunset at 18:40). During hours 16–24 (18:15 to 02:15), bats were likely to be active again. For further details of the proximity sensors, see Refs. 21, 23, and 29.

#### Long‐term cooperative relationships

Nine days after being introduced, the same bats were later used in an experiment that required observing their allogrooming for 16 weeks.^18^ The delay of 9 days was caused by a change in the method of data collection (the bats could not be reliably identified through infra‐red transmitting acrylic windows due to smudging of the acrylic). All bats were present in the flight cage during sampling weeks 1–6. Then experimenters randomly assigned 21 bats into 7 selected trios (one bat from each wild capture site) housed in small cages during week 7 (forced proximity treatment). Next, the experimenters allowed the bats to freely interact again in the flight cage during weeks 8–16. Throughout the study, experimenters did 682 h of all‐occurrence sampling^24^ using three stationary infrared surveillance cameras and then estimated hourly dyadic allogrooming rates. For further details, see Ref. 18.

#### Data analysis

To estimate how well first‐contact durations predicted long‐term allogrooming relationships, we fit Bayesian negative binomial multi‐membership models using the R package *brms*.^30^ Multi‐membership models account for interdependent undirected pairs being the property of both partners as random intercepts.^31^ Across all models, we used default priors, and we used posterior predictive checks to assess model fit, and Markov Chain Monte Carlo convergence was evident from Rhat values that ranged from 1.0001 to 1.0008 (across six chains), and Bulk and Tail effective sample sizes ranged from 9775 to 25,719.

To test our expectation that unfamiliar bats would spend less time in proximity than would familiar bats, we fit a negative binomial multi‐membership model with unfamiliarity as the binary predictor of minutes of contact. To assess the stability of our results, we fit six models for contact duration during sampling periods of 4, 8, 12, 16, 20, and 24 h. We also visualized contact networks to assess network density and assortativity based on capture site (Figure S1).

To model how well dyadic first‐contact durations predicted long‐term allogrooming relationships, we fit negative binomial multi‐membership models for unfamiliar pairs with first‐contact duration as a predictor of long‐term allogrooming rates. We again fit six models for first‐contact durations during sampling periods of 4, 8, 12, 16, 20, and 24 h to assess the stability of our results. To account for sampling biases across pairs, we modeled allogrooming rates using total minutes of observed dyadic allogrooming over 15 weeks as the response (overdispersed counts), and the total possible minutes of dyadic allogrooming as a log‐transformed offset term (exposure variable). To control for the effect of the forced proximity treatment on allogrooming rates, we also included whether the pair was forced into proximity (yes/no), whether the hourly grooming rate was estimated before versus after the treatment, and the interaction between those two effects. This interaction term represents the estimated causal effect of 1 week of forced proximity on the change in allogrooming rates, because the randomly assigned “forced proximity” treatment led to persistently higher allogrooming rates in the treated bat pairs relative to control pairs.^18^ We therefore used the interaction terms from these models (i.e., the increase in allogrooming associated with 1 week of forced proximity) as a reference effect to compare against our main result of interest (the increase in allogrooming rate associated with a one‐standard‐deviation increase in first‐contact duration).

After our main analysis, we conducted a post hoc analysis to investigate whether first‐contact durations were more successful at predicting allogrooming rates when only including allogrooming data that were collected before the forced proximity treatment. Because these allogrooming events also occurred closer in time to the first contacts, we expected this analysis to greatly increase our power to detect any predictive role for first contacts. We fit the same kind of model described above except that first‐contact duration was now the only predictor of allogrooming rates over the 6 weeks prior to the forced proximity treatment in week 7 (we did not include forced proximity as a predictor because it had not occurred yet).

To estimate and visualize the proportion of variance explained by first‐contact durations across all models, we computed a Bayesian version of *R* ^2^ for the full model in *brms* ^30^ following the method by Gelman et al.^32^ We then computed the *R* ^2^ value for the models without the first‐contact term, and we used the difference as the proportion of variance explained by the first contacts.

#### Sensitivity analysis

In addition to fitting models for six possible sampling periods (4, 8, 12, 16, 20, and 24 h), we also tested the robustness of our conclusions another way. We repeated the entire analysis described above using only the top 1% (rather than the top 5%) of maximum signal strength values to define “close contacts”. Compared to contacts, close contacts lead to fewer observations per pair and lower social network density (fewer connected pairs), but they should be an even better proxy for clustering and allogrooming because they correspond to an expected distance of only about 2 cm.^23^ We mainly report these alternative analyses (using close contacts) in the Supporting Information section, because the results were similar and the conclusions were the same.

### Results

#### Ingroup biases in contact

**FIGURE 2:**
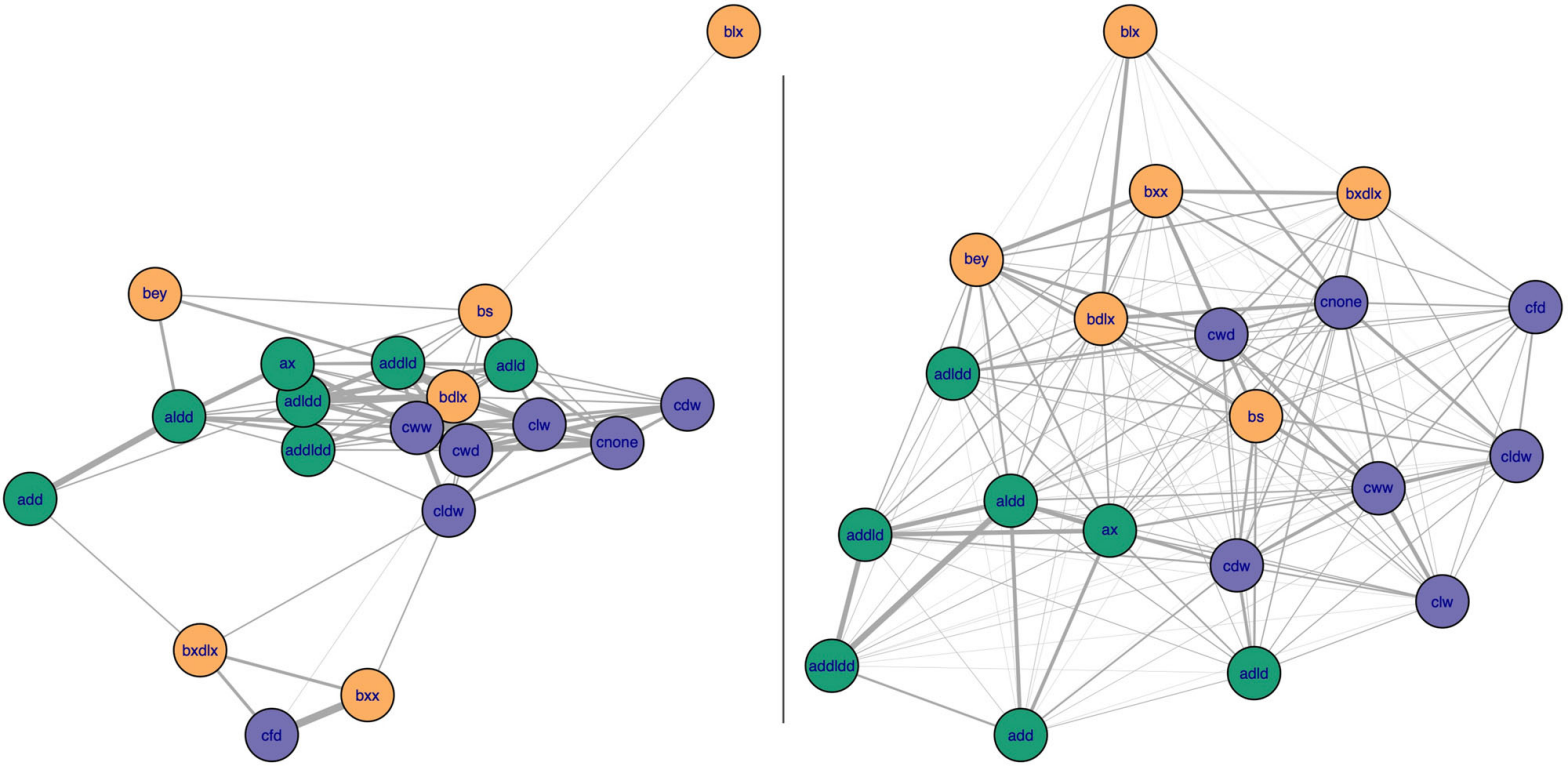
Contact networks show ingroup association bias after 4 and 24 h. Contact networks for bats (nodes) captured from three field sites (colors) and placed together in a flight cage after 4 h (left) and 24 h (right). Network edges (links) are proportional to contact durations, and edges between bats of different colors are first‐contact durations. Graph layouts use the Fruchterman and Reingold algorithm implemented by the *igraph* R package. The full set of contact networks for 4, 8, 12, 16, 20, and 24 h are shown in Figure S1. The full set of close‐contact networks for 4, 8, 12, 16, 20, and 24 h are shown in Figure S2.

**FIGURE 3:**
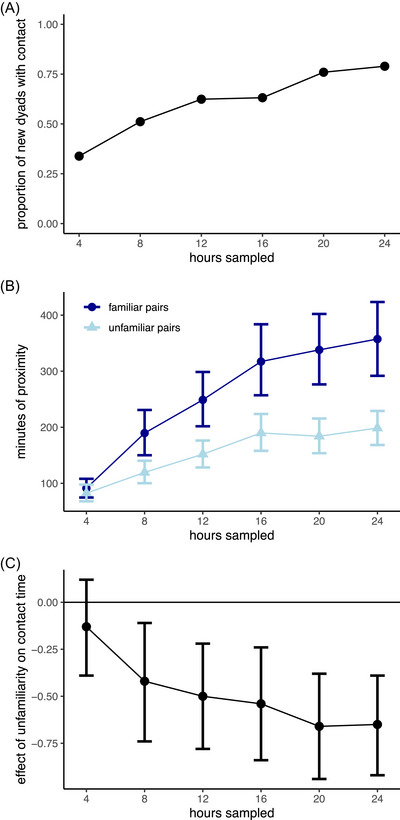
The effect of familiarity on contact rates became increasingly evident with increased sampling over time. Panel A shows the proportion of unfamiliar pairs in contact over time. Panel B shows the mean and bootstrapped 95% confidence interval for minutes of proximity between familiar pairs (dark circles) and unfamiliar pairs (light triangles). Panel C shows the posterior probability of the coefficient for the effect of unfamiliarity on contact times with 95% Bayesian credible interval.

Over the initial 24‐h period of contact tracking, the female vampire bats showed clear in‐group preferences (Figure 2). These preferences became more evident as the sample size of first contacts increased from 45 of 133 pairs of unfamiliar bats (34%) within the first 4 h, to 105 of 133 pairs (79%) within 24 h (Figure 3A). As expected, bats from the same capture site spent more time in contact than did unfamiliar pairs, but this difference did not emerge until after sunrise (Figure 3B). The first 4 h of contact data therefore suggest a high degree of social mixing and possible social exploration, followed by a clear bias toward roosting with familiar bats (Figure 3C). We observed the same pattern when only analyzing the smaller sub‐sample of close contacts (Figure S3). Despite this ingroup contact bias, we saw that bats were willing to roost in the same spaces as previously unfamiliar conspecifics. Although the three groups of bats were given access to three roost boxes, each group did not clearly occupy a separate box on the next morning after the introduction.

#### First contacts and long‐term bonds

**FIGURE 4:**
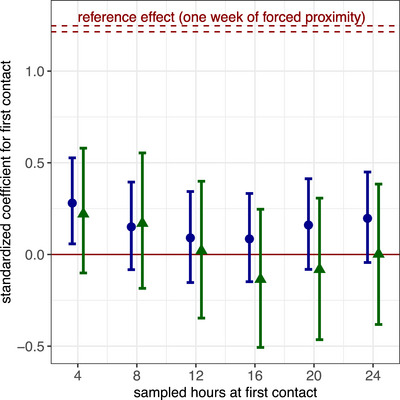
First‐contact durations do not strongly predict long‐term allogrooming relationships. Means and error bars show the standardized coefficient estimates (points) and 95% Bayesian credible intervals (error bars) for first‐contact durations, during the first 4–24 sampled hours, as a predictor of allogrooming rates, averaged over either all 15 sampled weeks (blue circles) or the first six sampled weeks (green triangles). Estimates are nudged horizontally slightly to avoid overlap. Reference lines (dashed red) show the minimum and maximum coefficient for the effect of one week of forced proximity on long‐term allogrooming.

Overall, the ability for first‐contact durations to predict long‐term allogrooming relationships was weak (Figure 4). Across all estimates, the highest statistical association between first contacts and long‐term bonds occurred for the first 4 h of short‐term first contact, when a one‐standard‐deviation increase in dyadic first‐contact duration predicted a 32% increase in dyadic long‐term allogrooming rates (Table S2). However, this association was small relative to the effect of a 1‐week period of forced proximity, which caused a 238% increase in long‐term allogrooming rates. Moreover, the correlation diminished with increased sampling: After 16 h, a one‐standard‐deviation increase in contact time was associated with only an 8% increase in allogrooming rates, and the Bayesian 95% credible interval for the posterior probability of all five other coefficients from hours 8–24 included zero and negative values (Figure 4, Table S2). In other words, a greater sampling of first‐contact duration led to it being a worse and less certain predictor of future long‐term cooperative relationships.

The ability of first close contacts to predict long‐term allogrooming relationships was even weaker (Figure S4). In this case, the increase in dyadic long‐term allogrooming rates associated with a one‐standard‐deviation increase in dyadic first‐contact duration during the first 4 h fell to 16%, and the Bayesian 95% credible intervals for this and all other estimates included zero and negative values.

If first‐contact duration does predict long‐term allogrooming, then it should be a better predictor for allogrooming rates that were (1) closer in time to the first contacts and (2) measured prior to the experimental manipulations (forced proximity treatment). Instead, we found the opposite result: First‐contact durations were worse at predicting the earliest allogrooming rates measured over the first 6 weeks (Figure 4, Table S3). These earlier allogrooming rates were also not clearly predicted by first close‐contacts (Figure S4).

**FIGURE 5:**
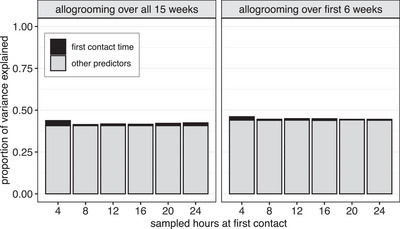
First‐contact duration explained only 0.6%–3% of the variance in allogrooming. Height of bars shows the proportion of variance in long‐term allogrooming explained by the full model (*R* ^2^), including the proportion explained by first‐contact durations (black) and the rest of the model (gray).

Across all models, first‐contact duration explained almost none of the variance in allogrooming rates (Figure 5). Full models explained 41.4%–46.0% of variance in long‐term allogrooming rates, but first‐contact durations explained only 0.56%–2.9%, whereas the other model terms collectively explained 40.7%–44.0% (Figure 5). Furthermore, first close‐contacts explained <1.2% of the variance in allogrooming rates (Figure S5). In sum, the ability of first‐contact durations to predict long‐term allogrooming was both imprecise and low.

## DISCUSSION

Long‐term cooperative relationships among female vampire bats were not highly predictable from how the two bats interacted during their first encounter. This finding shows that relationship outcomes are not entirely a product of each individual's immediately observable phenotypic traits such as their body size, aggressiveness, tolerance, scent, vocalizations, or their immediate social interactions at first encounter. All these factors may have influenced long‐term relationships, but they did not clearly predict which relationships would form. Therefore, our results reject the immediate assessment hypothesis and support the social history hypothesis, which assumes that the effects of immediately observable phenotypic traits on social bonding can be overshadowed either by a longer period of social assessment or by changes in social preferences.

There are several caveats to keep in mind when interpreting our results. First, our statistical power only allowed us to reject the immediate assessment hypothesis, a null hypothesis that assumes no role of social history. Although this is an important first step, the ultimate long‐term goal for understanding social relationship formation is to more precisely measure the relative importance of individual and relationship traits over time as new relationships form and as social networks stabilize. This effort will require tracking a much larger sample of individuals as they form new relationships. Second, our results do not show that immediate assessments or first interactions play no causal role in social bonding. Rather, our findings are consistent with the assumption that social information gathered by each individual during first contacts is likely updated continuously during the process of relationship development. Finally, in study 1, the bats could interact vocally and possibly through olfaction before they first met, so it was possible that they formed impressions of each other through vocal or chemical interactions before their first physical encounters. Although this possibility does not change our conclusions that first contacts do not predict long‐term bonds, we are now conducting studies on the roles of vocal interactions in social bonding in this species to address it further.

The disconnect between initial interactions and long‐term relationships will not be a surprise for readers who already assume that social bonding is a process responding to complex and unpredictable social network dynamics. However, testing these assumptions remains important because many seemingly complex social interactions can result from surprisingly simple heuristics. For example, a series of experiments showed that rats will repeatedly help a partner (by pulling a sliding tray) when that partner had reciprocally groomed them or fed them using the same or a different food‐delivery apparatus.^33^, ^34^, ^35^ However, rather than integrating their experiences of help over time with each partner, they simply seemed to match the partner's most recent behavior.^36^ Later experiments showed that the odor cues from a partner pulling the food tray were sufficient to induce helping behavior in the focal subject, even in the absence of help from the partner.^37^ In other words, the scent of a partner pulling the tray was more important for helping decisions than the actual experience of receiving food on the tray.^37^ Such results highlight the importance of considering simple heuristics when interpreting the causes of helping and other social behaviors.

Several lines of evidence do suggest that female vampire bats integrate experiences both over time and across partners. They appear to “test the waters” when forming new nonkin relationships in the way they transition from clustering to allogrooming to food sharing.^18^, ^19^ Nonkin cooperative relationships persist from captivity to the wild,^23^ and from the roost to a foraging context.^21^ The ability for individuals to cope with the temporary removal of a food donor appears to depend on the number of food‐sharing relationships they have built with nonkin.^38^ Agent‐based models of social bonding in vampire bats suggest that individuals must balance the need to strengthen existing relationships and invest in building new ones.^12^, ^39^ This trade‐off between the benefits of building new cooperative relationships and avoiding the risks of contact with unfamiliar strangers is likely to shape their behaviors with strangers.

The first‐contact behaviors we observed among vampire bats were largely affiliative. What explains the low amount of aggression between strangers? The extent to which strangers pose a threat versus an opportunity is likely to vary greatly with sex and species. Female vampire bats appear to have some of the most egalitarian and the least consistent dominance relationships among female mammals.^40^ Aggression is clearly higher among male vampire bats and males appear to form more clear dominance relationships.^27^, ^41^ Our findings about female vampire bats are therefore unlikely to generalize to males due to their higher rates of aggression and their lower rates of affiliation, allogrooming, and food sharing toward both other males and females.^20^, ^27^, ^41^, ^42^

A general prediction is that the ratio of expected threat to expected benefit is likely to affect how animals from different species or populations evaluate strangers and that more risky interactions are likely to necessitate longer periods of initial assessment. For species that live in closed, stable groups and where most resource competition is between groups (e.g., cooperative breeders), interactions with familiar groupmates pose high benefits and low risk (e.g., infectious disease and aggression), whereas interactions with strangers pose low benefits and high risks.^43^ In contrast, for species that live in societies with more fluid fission–fusion social dynamics^44^ (including vampire bats^27^, ^45^), the differences between familiar and unfamiliar females may not be as stark, and strangers that join a group might be potential cooperation partners.^19^ In vampire bats, little is known about rates of intergroup aggression. In humans, however, where intergroup conflict is well documented, there is a surprising tendency to help strangers, which is common across age groups and across different human societies, and which has been hypothesized to exist because strangers in these studies might be seen as potential future cooperation partners.^46^, ^47^, ^48^, ^49^, ^50^ In our closest primate relatives, the ratio of risk to benefit for interacting with strangers seems to vary with the amount of intergroup aggression. In chimpanzees, intergroup encounters can lead to aggression, and participation in these risky collective actions are influenced by within‐groups social bonds.^51^ The xenophobia commonly observed in chimpanzees is therefore likely explained by a local scale of cooperation, and a larger scale of competition, leading to the threat of lethal intergroup aggression, whereas bonobos have repeatedly demonstrated xenophilia, that is, helping behavior toward unfamiliar individuals, which is hypothesized to be explained by a greater potential of new cooperative relationships with strangers and a history of sexual selection for reduced male aggression.^48^

Within a species, social bonding and behavior toward strangers might be predicted by the need of different individuals to either extend or maintain their existing social relationships. For instance, wild jackdaws preferentially built co‐foraging relationships with individuals that provided greater returns, but this relationship‐building appeared to be constrained by a need to maintain existing close social relationships.^52^ In baboons and great tits, individuals that recently lost a close social partner were more likely to engage in behaviors that build new relationships.^53^, ^54^ The importance of first impressions for the trajectory and success of cooperative relationships should be particularly important in cases where there is competitive partner choice that selects for cooperative traits, as in humans.^13^, ^55^

The initial interactions between strangers can provide insights into other kinds of long‐term social relationships beyond cooperative (friendship‐like) relationships. For example, prior to the formation of reproductive pair bonds, subtle changes in associations before the reproductive period might reflect how individuals sample potential partners to choose mates.^56^ In such cases, the importance of first impressions versus long‐term partner assessment is likely to depend on factors such as the relative importance of fixed phenotypic traits versus cues to the partner's parental care ability^57^ or compatibility. For example, in a study of pair‐bonding in titi monkeys (*Plecturocebus cupreus*), experimenters measured initial interactions among six male and six female unpaired adults across six 30‐min interaction periods to assess their initial compatibility, they then used these first interaction rates to create six male–female test pairs of highest compatibility.^58^ Compared to 13 matched control pairs, the 6 test pairs with higher initial compatibility developed stronger pair bonds as evident from rates of intertwining their tails.^58^

The role of first impressions can also vary for long‐term dominance relationships, depending on the relative importance of fixed traits versus social experience. For example, in golden‐crowned sparrows (*Zonotrichia atricapilla*), manipulations of an individual's phenotype caused strangers to react to the manipulated signal, but familiar individuals did not change their behavior toward the manipulated bird.^59^ In the absence of social experience (i.e., interactions with a stranger), individuals based their social behaviors on first impressions but the alterations to these phenotypes (dominance signals) were disregarded when there was a social history.

In conclusion, our results are consistent with the hypothesis that the formation of cooperative relationships in vampire bats does involve integration of social interactions over time. This process of social integration is often hypothesized to be similar to a process of associative learning or Bayesian updating.^12^, ^60^, ^61^ However, surprisingly little is known about how animals incorporate social events over time, how they learn about and compare potential partners and rivals, and on what timescale different phases of social bonding occur in nonhuman animals. We suggest that studies tracking and manipulating both the formation and maintenance of new relationships at high resolution will be essential to a further understanding of the causes and consequences of social integration.

## AUTHOR CONTRIBUTIONS

**Gerald G. Carter**: Conceptualization; methodology; software; formal analysis; investigation; resources; data curation; writing—original draft; visualization; supervision; project administration; funding acquisition. **Simon P. Ripperger**: Methodology; software; formal analysis; investigation; resources; data curation; writing—review and editing. **Vi Girbino**: Investigation; writing—review and editing. **Imran Razik**: Investigation; writing—review and editing. **M. May Dixon**: Validation; writing—review and editing. **Rachel A. Page**: Resources; supervision; project administration; writing—review and editing. **Elizabeth A. Hobson**: Writing—review and editing; funding acquisition. Credit taxonomy from https://credit.niso.org/

## CONFLICT OF INTEREST STATEMENT

The authors declare no conflicts of interest.

### PEER REVIEW

The peer review history for this article is available at: https://publons.com/publon/10.1111/nyas.15241

## Supporting information



Supporting information

## Data Availability

Data and R code are available on Figshare (https://doi.org/10.6084/m9.figshare.25996702.v1).
