# Supplementary material for: Long‐term cooperative relationships among vampire bats are not strongly predicted by their initial interactions
Source: Ann N Y Acad Sci. 2024 Oct 27;1541(1):129–39. doi: 10.1111/nyas.15241 (PMC11580772; doi:10.1111/nyas.15241)
Supplement: Supplementary file 1 — Supporting information [file NYAS-1541-129-s001.docx]

Supporting Information for

**Long-term cooperative relationships among vampire bats are not strongly predicted by their initial interactions**

Gerald G. Carter^1,2^, Simon P. Ripperger^3^, Vi Girbino^1^, M. May Dixon^1^, Imran Razik^1,2^, Rachel A. Page^2^, Elizabeth A. Hobson^4^

**Affiliations**

^1^ Department of Evolution, Ecology and Organismal Biology, The Ohio State University, Columbus, Ohio, USA;

^2^ Smithsonian Tropical Research Institute, Apartado 0843-03092 Balboa, Ancón, Panamá

^3^ Museum für Naturkunde, Leibniz-Institut für Evolutions- und Biodiversitätsforschung, Invalidenstraße 43, 10115 Berlin, Germany

^4^ Department of Biological Sciences, University of Cincinnati, Cincinnati, Ohio, USA

**Correspondence:** Gerald Carter, Department of Ecology and Evolutionary Biology, Princeton University, Princeton, NJ, USA. Email: gc1511@princeton.edu

**Table S1. Permutation test results.**

| Long-term relationship | Predictor interactions | Observed coefficient | Expected null coefficient | Null lower 95% quantile | Null upper 95% quantile | One-tailed P |
| --- | --- | --- | --- | --- | --- | --- |
| allogrooming | affiliative | 0.930 | 0.589 | 0.192 | 0.948 | 0.07 |
|  | aggressive | -0.402 | -0.327 | -1.080 | -0.092 | 0.24 |
| food sharing | affiliative | -0.443 | -1.704 | -5.224 | -0.308 | 0.19 |
|  | aggressive | -0.370 | -2.703 | -4.389 | 0.414 | 0.72 |

**Table S2. Coefficients for first-contact durations (scaled) as a predictor of long-term allogrooming relationships.** Columns are: number of hours sampled, coefficient estimate for first-contact duration, error estimate, upper and lower credible intervals, R-hat, Bulk and Tail Effective Sample Sizes, R^2^ for the full model, and R^2^ for the model without first contact duration included. These estimates are plotted in Figure 4.

| Sampled hours | Coeff Est | Error Est | lower 95% CI | upper 95% CI | Rhat | Bulk ESS | Tail ESS | R-squared | R-squared null |
| --- | --- | --- | --- | --- | --- | --- | --- | --- | --- |
| 4 | 0.280 | 0.120 | 0.058 | 0.527 | 1.000 | 14777 | 11765 | 0.438 | 0.407 |
| 8 | 0.151 | 0.123 | -0.083 | 0.395 | 1.000 | 14286 | 13130 | 0.414 | 0.408 |
| 12 | 0.090 | 0.127 | -0.153 | 0.343 | 1.000 | 13266 | 13197 | 0.418 | 0.408 |
| 16 | 0.085 | 0.124 | -0.149 | 0.333 | 1.000 | 13610 | 13128 | 0.416 | 0.408 |
| 20 | 0.160 | 0.126 | -0.081 | 0.413 | 1.000 | 14024 | 12309 | 0.421 | 0.407 |
| 24 | 0.197 | 0.125 | -0.044 | 0.450 | 1.000 | 13884 | 12567 | 0.424 | 0.408 |

**Table S3. Coefficients for first close-contact durations (scaled) as a predictor of long-term allogrooming relationships.** Columns are: number of hours sampled, coefficient estimate for duration of first close contact, error estimate, upper and lower credible intervals, R-hat, Bulk and Tail Effective Sample Sizes, R^2^ for the full model, and R^2^ for the model without first close-contact duration included. These estimates are plotted in Figure S4.

| Sampled hours | Coeff Est | Error Est | lower 95% CI | upper 95% CI | Rhat | Bulk ESS | Tail ESS | R-squared | R-squared null |
| --- | --- | --- | --- | --- | --- | --- | --- | --- | --- |
| 4 | 0.221 | 0.173 | -0.101 | 0.580 | 1.000 | 14862 | 12239 | 0.461 | 0.441 |
| 8 | 0.170 | 0.187 | -0.185 | 0.554 | 1.000 | 14023 | 12869 | 0.446 | 0.439 |
| 12 | 0.021 | 0.190 | -0.347 | 0.399 | 1.000 | 12692 | 13404 | 0.449 | 0.440 |
| 16 | -0.135 | 0.190 | -0.507 | 0.247 | 1.000 | 12432 | 12004 | 0.449 | 0.439 |
| 20 | -0.081 | 0.197 | -0.465 | 0.307 | 1.000 | 12989 | 12865 | 0.445 | 0.442 |
| 24 | 0.002 | 0.196 | -0.382 | 0.384 | 1.000 | 11828 | 13010 | 0.445 | 0.440 |

**Figure S1. Contact networks over time show stability of ingroup bias.** Contact networks for bats (nodes) captured from three field sites (Lake Bayano: green, Tolé: orange, La Chorrera: violet) and placed together in a flight cage for 4, 8, 12, 16, 20, and 24 hours. Network edges (links) are proportional to contact duration. Edges between bats of different colors are first contact durations. Graph layouts use the Fruchterman and Reingold algorithm implemented by the igraph R package.


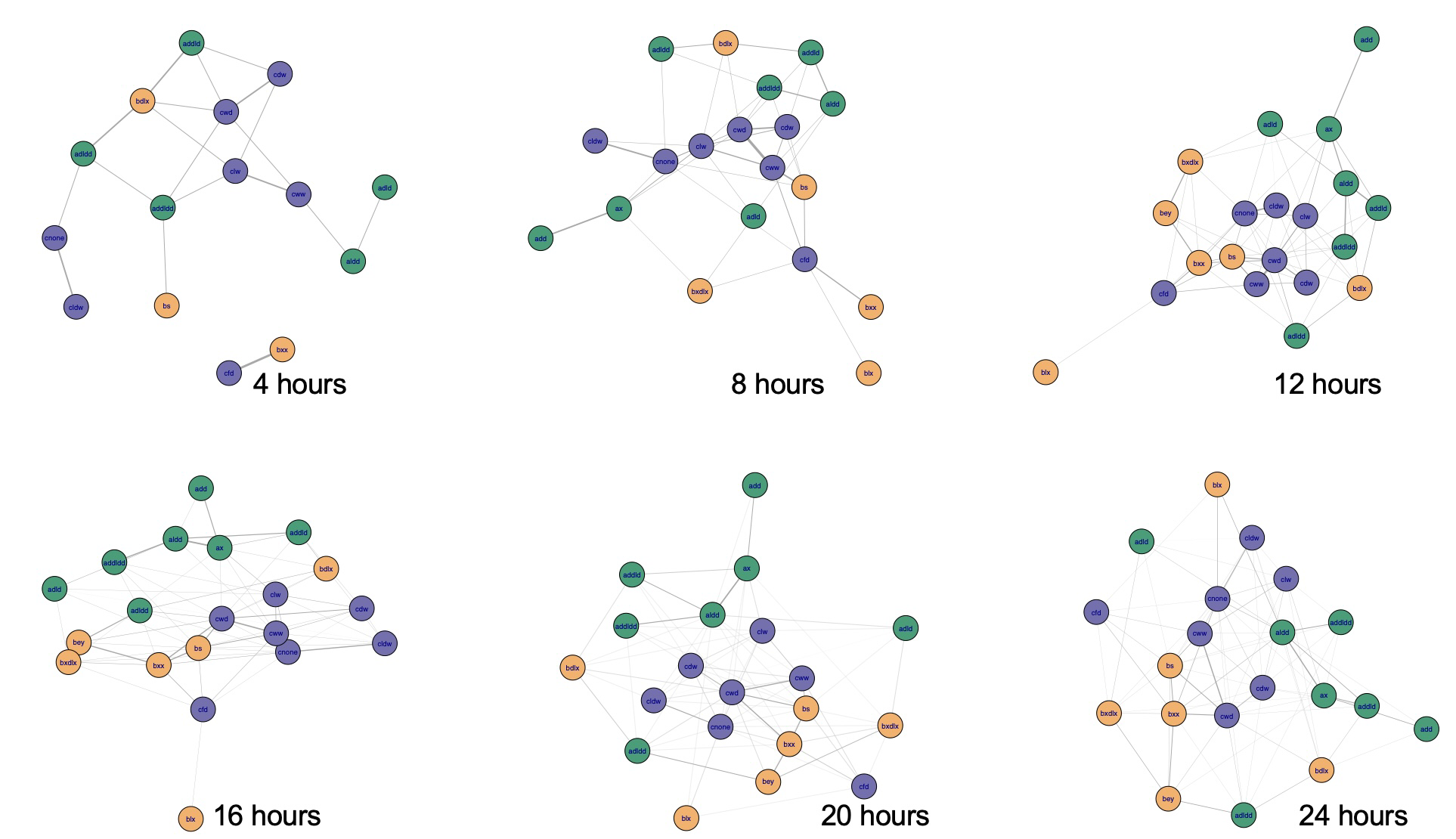


**Figure S2. ‘Close-contact’ networks (top 1% of proximity values) show less dramatic assortment than contact networks (top 5% of proximity values) but stability of ingroup bias remains.** Close-contact networks for bats (nodes) captured from three field sites (Lake Bayano: green, Tolé: orange, La Chorrera: violet) and placed together in a flight cage for 4, 8, 12, 16, 20, and 24 hours. Network edges (links) are proportional to contact duration (about 2 cm proximity). Edges between bats of different colors are first close-contact durations. Graph layouts use the Fruchterman and Reingold algorithm implemented by the igraph R package.


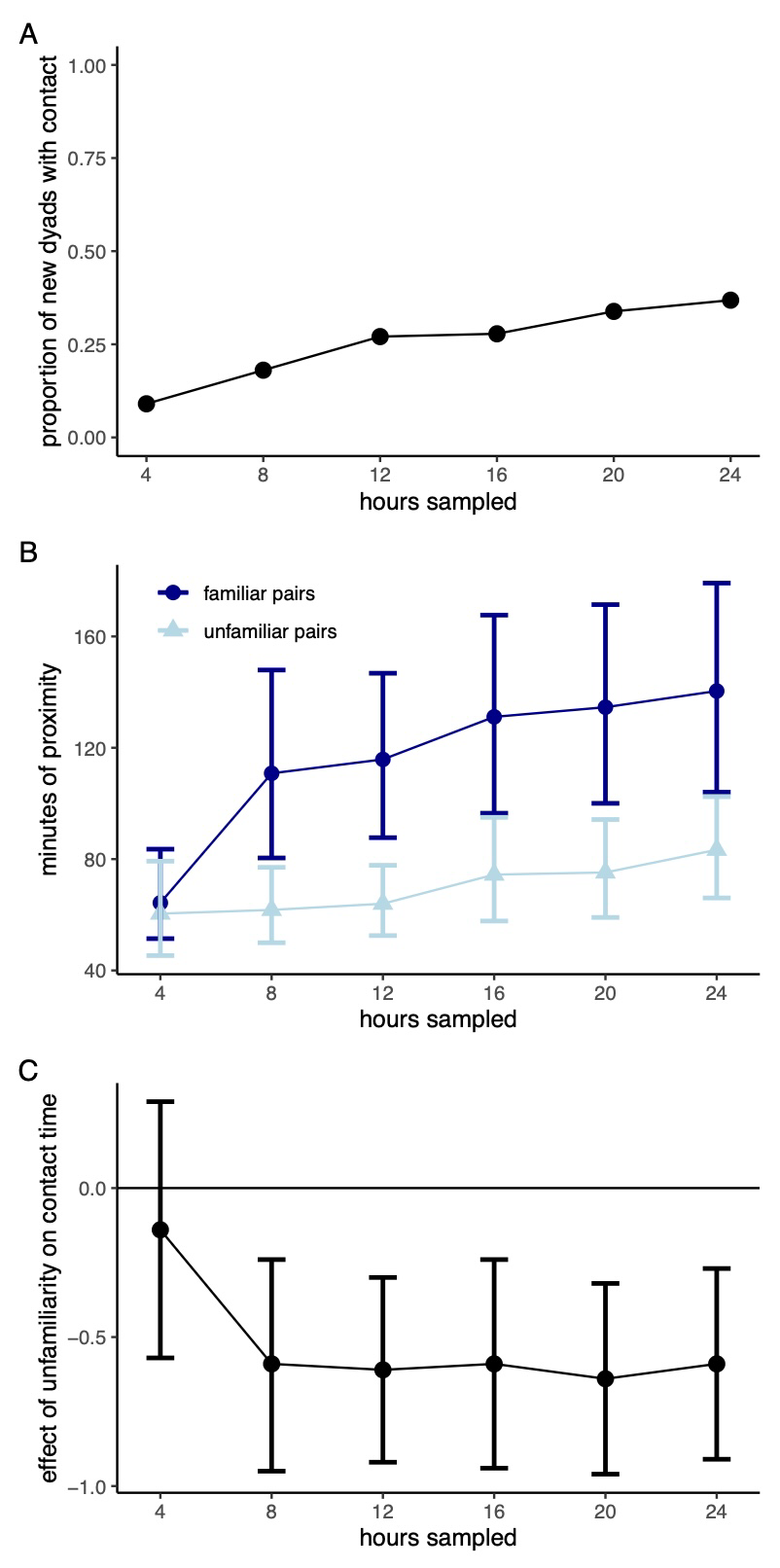
**Figure S3. The effect of familiarity on ‘close-contact’ rates became increasingly evident with increased sampling over time.** Panel A shows the proportion of unfamiliar pairs in contact over time. Panel B shows the mean and bootstrapped 95% confidence interval for minutes of proximity between familiar pairs (dark circles) and unfamiliar pairs (light triangles). Panel C shows the posterior probability of the coefficient for the effect of unfamiliarity on contact durations with 95% Bayesian credible interval.

**Figure S4. First close-contact durations do not strongly predict long-term allogrooming relationships.** Means and error bars show the standardized coefficient estimates (points) and 95% Bayesian credible intervals (error bars) for first contact durations, during the first 4 to 24 sampled hours, as a predictor of allogrooming rates, averaged over either all 15 sampled weeks (blue circles) or the first 6 sampled weeks (green triangles). Estimates are nudged horizontally slightly to avoid overlap. Reference lines (dashed red) show the minimum and maximum coefficient for the effect of one week of forced proximity on long-term allogrooming.

**Figure S5. First close-contact duration explained only 0-1% of the variance in allogrooming.** Height of bars show the proportion of variance in long-term allogrooming explained by the full model (R^2^), including the proportion explained by first contact durations (black) and the rest of the model (grey).
